# Who is teaching in Chinese primary schools? A profile of the primary education workforce in Chinese county areas

**DOI:** 10.1371/journal.pone.0245549

**Published:** 2021-01-19

**Authors:** Ming Huo, Na Zhao, Yue Zhao, Wim Van Den Noortgate

**Affiliations:** 1 Faculty of Education, Northeast Normal University, Changchun, China; 2 Faculty of Psychology and Educational Sciences & imec-ITEC, KU Leuven, Kortrijk, Belgium; Institute for Advanced Sustainability Studies, GERMANY

## Abstract

A teaching workforce with good quality is a key factor in the process of China’s rapid development. Although 76% of Chinese pupils are studying at schools within county areas, a general portray of the corresponding teaching workforce is still not clear. This study presents data from a nationally representative survey of primary education teachers in 35 counties of 18 provinces in China. Findings presented include demographic and professional characteristics, living conditions as well as attitudes towards work. Besides, variations among school locations and geographical regions are also examined. The key findings are the followings: 1) Quality of primary school teachers in county areas has been improved regarding education background; 2) Teaching force in village primary schools has an unbalanced age and gender composition; 3) Out-of-field teaching practice is widespread, especially for minor subjects. 4) Primary school teachers perceived relative low salary and low social status.

## Introduction

That education plays a vital role in the economic growth of a nation is recognized for decades [1]. Various subsequent studies found a positive impact on economic growth of educational enrolment and attainment [2–4] and an increasing role of education in cognitive skills’ development [5, 6]. Researchers have even argued that a long run economic growth may not be sustained without the improvement of nationwide educational achievement [7, 8].

A key factor in raising educational achievement is teacher quality [9–13]. A recent study on OECD (The Organisation for Economic Co-operation and Development) countries has shown that an increase of one standard deviation in teacher cognitive skills is associated with an increase of 10 to 15 per cent of a standard deviation in student performance [14].

Newly released PISA (Programme for International Student Assessment) result showed that students from mainland China were ranked very high in this global survey. However, due to the fact that only four Chinese areas were surveyed and these belong to the wealthiest areas, the results cannot be regarded as a portrait of nation-level student performance. The successful transition towards a nation with strong human resources depends much more on the improvement of student achievement in the vast county-level areas. A high quality teaching workforce is the key to the construction of a high-level compulsory education in county areas.

China has an enormous teaching workforce to serve the largest school-age population in the world [15]. The teaching workforce in Chinese primary schools consists of around 5.3 million full-time teachers across the whole nation, among which around 80% are working in the county-level schools and are responsible for the teaching of 76% primary school students nationwide [16]. To enhance the quality of primary teaching workforce in county-level schools, one important aspect is an accurate monitoring of teachers’ demographic, professional characteristics, living conditions, attitudes towards work, and problems experienced by the teacher workforce, accounting for inter-school and inter-regional differences. Overall, deepening our understanding of county-level teachers’ characteristics has important implications as to how the teaching workforce can be supported, be improved, and serve as a driving force of China’s human resource transformation.

Although the literature provides some information about the population of primary teachers working in the county areas of China [17–26], prior studies are only concentrating on certain topics such as teachers’ salary [17], rank [18, 23], current characteristics [19, 26], policy evaluation [20], psychological well-being [20, 21, 22, 24, 25], etc. There is no study providing comprehensive information about primary teaching workforce in county-level schools. The goal of this study is to fill this gap, to have a baseline measurement of the aforementioned variables that can serve in the future to monitor evolution.

In view of this, we conducted a questionnaire survey on approximately 37,000 primary teachers from 35 counties in 18 provinces with respect to four perspectives: teachers' demographic and professional characteristics, living conditions, and attitudes towards work.

## Methodology

### Context

To have a better understanding of the design and results of this study, we provide a brief introduction to the educational system, the system of teacher education and certification, and the administrative divisions of China.

#### The educational system in China

In China, compulsory education lasts for nine years and children start school at the age of six. Compulsory education can be divided into two parts: primary education and lower secondary education. Primary education is aimed for children from six to twelve and consists of six consecutive years of study (grades 1–6). Lower secondary education is intended for young people aged twelve to fourteen and organized in three consecutive years (grades 7–9). At the upper secondary education stage (grades 10–12), two educational types can be distinguished based on their educational aims, namely: general education and vocational education. Although upper secondary education is not compulsory, most (89%) Chinese young people are enrolled [16]. Responsibility for primary, lower secondary, and upper secondary education rests with the governments at the county level.

#### Teacher education and certification

Before 1999, a three-level teacher education system was organized to provide teaching training. This system included three types of hierarchical institutions: “normal schools” (*zhongshi*) aiming to train primary school teachers and award a diploma similar to upper secondary education, “professional teacher colleges” (*gaodengshizhuan*) training teachers for lower secondary education and awarding an associate degree, and normal universities and colleges (*shifandaxue*) training senior secondary school teachers and awarding a bachelor degree. In order to fulfil the demand of high quality education and improve quality and effectiveness of teacher education, the Ministry of Education in 1999 presented the document “Suggestion on Restructuring the Teacher Education Institutions” to reorganize the old three-level teacher institution system of normal school, professional teacher college, and normal university into a two-level system of professional teacher colleges and teacher colleges or normal universities, where all new elementary and secondary school teachers would be prepared by 2010 [27]. Under the guidance of this document, all normal schools merged with professional, teacher colleges, grew into professional teacher colleges, or transformed into secondary schools [28]. In addition, some professional teacher colleges upgraded to four-year teacher colleges. Apart from the above actions, some comprehensive universities and colleges were also recommended to establish teacher education programs. Since then, the monopoly of teacher education by independent teacher education institutions broke and a free market of teacher supply has been built [29].

Before 1995, there were no teacher certificate examinations in China. In most cases, only graduates from normal schools, professional teacher colleges, or normal universities could become primary or secondary school teachers. Normally these graduates automatically became teachers when assigned to schools by the government. On December 1995, “Regulations on Teacher Certification” was issued by the State Council requiring that the teacher candidates not graduating from teacher education institutions pass the teacher certification exam to become a teacher [27]. These exams were organized in the provincial level and composed of two main fields: pedagogy and psychology. All candidates who passed the exams would be offered a permanent Teacher Certificate and was recognized nationwide. Since 2011, China has gradually promoted the national teacher qualification examination system piloting in eight provinces. From 2013, the national Teacher Qualification Examination was implemented in all provinces. At present, the only way to become a teacher in China is to pass the national exam and get the Teacher Certificate.

#### Administrative divisions of China

China’s administrative system comprises a nested hierarchy of authorities [30]. Under the central government, in a descending order, there exist four levels of sub-national government: province (*shengji*), prefecture (*diji*), county (*xianji*), and township (*xiangji*). These sub-national governments also appear in different varieties and forms as shown in Table 1. Below these four administrative levels, a fifth level, “village level” (*cunji*), also exists. Although not considered a level of government, the administrative village performs a number of important functions for hundreds of millions of Chinese rural residents [31]. County-level and township-level governments can be further divided into urban and rural manifestations. For instance, urban districts (*qu*) and county-level municipalities (*xianji shi*) are urban manifestations at the county-level units and urban sub-districts (*jiedao*) are urban manifestations at the township-level. Counties and autonomous counties are rural manifestations at the county-level, while towns (*zhen*) and townships (*xiang*) are rural township-level units (towns tend to be more urbanized and industrialized than townships). Besides, at the village level, administrative villages (*xingzheng cun*) are units in rural areas, while neighbourhood committees (*shequ*) are urban units [31].

**Table 1:** Structure of China’s administrative divisions (Ministry of Civil Affairs, 2018).

Tier	Level	Form and quantity	Total Number across China
1	Province-level	Provinces: 23	34
		Centrally administered municipalities: 4	
		Ethnic minority autonomous regions: 5	
		Special Administrative Regions: 2	
2	Prefecture-level	Prefecture-level municipality: 293	333
		Autonomous prefecture: 30	
		Prefecture:7	
		League:3	
3	County-level	Urban district: 970	2,851
		County-level municipality: 375	
		County: 1,335	
		Autonomous county: 117	
		Banner/autonomous banner: 52	
		Special district: 1	
		Forestry area: 1	
4	Township-level	District public office: 2	39,945
		Town: 21,297	
		Township: 9,118	
		Ethnic township: 1,135	
		Urban sub-district: 8,393	
NA	Village-level	Administrative village	600,000+
		Neighborhood committee	

In this study, we focus on the teachers working in schools within counties. The area of a regular county in China usually consists of a number of towns or townships, and the capital town in which the county government is located is called a county seat. Except the county seat, a town or township usually consists of a number of villages. The capital village, where the town government is located, is called a town seat. The primary schools in a county are distributed in the county seat, town seats, and (other) villages. For simplicity, schools located in county seats, towns seats, and villages will be called later in this paper “county schools”, “town schools”, and “village schools”. Teachers working in these types of primary schools will be referred as “county teachers”, “town teachers”, and “village teachers” respectively.

### Participants

This study used a part of the larger data of National Survey of Teaching Workforce in County Areas conducted by the China Institute of Rural Education Development in 2018. The full samples of the survey consisted of teachers in preschools, primary schools, lower secondary schools, and upper secondary schools. This study used data from primary school teacher samples only.

Considering Chinese regional diversity in terms of geographical and economic development level, this survey randomly selected 18 provinces: Liaoning, Shandong, Fujian, and Guangdong in Eastern China; Shanxi, Henan, Anhui, Jiangxi, Hubei, and Hunan in Central China; and Gansu, Ningxia, Shaanxi, Sichuan, Chongqing, Guizhou, Yunnan, and Guangxi in Western China. For each province (except Hubei), two counties with different economic levels were selected, for a total of 35 counties. In every county, half of all towns with different economic levels were selected. In every town, a list of all primary schools was provided by the local government, and all full-time primary teachers working in these schools were invited to voluntarily complete an anonymous web-based questionnaire via *wenquexing*, a professional on-line survey platform (www.wjx.cn). In each school, a single contact person took responsibility for providing the information regarding the objectives of the study, distributing the questionnaire via Internet or an instant messaging application, and reporting the number of responding teachers to the research group. The questionnaires were answered online between April 2018 and July 2018. Of the 43,441 teachers from 2,993 primary schools on the name list were invited to participate in this survey, 36,274 primary teachers from 2,919 schools completed the questionnaires, resulting in an average response rate of 83.5%. (see Table 2 for the distribution of participating teachers among provinces and counties).

**Table 2:** Geographical distribution of the teacher sample.

Province	County	N	%	Province	County	N	%
Anhui	Jinzhai	757	2.09	Jiangxi	Dingnan	709	1.95
	Taihe	1,323	3.65		Xiushui	1,811	4.99
Fujian	Changtai	391	1.08	Liaoning	Jianping	932	2.57
	Gutian	430	1.19		Liaoyang	641	1.77
Gansu	Kang	343	0.95	Ningxia	Pengyang	651	1.79
	Lintao	960	2.65		Zhongning	771	2.13
Guangdong	Haifeng	1,483	4.09	Shandong	Cao	2,383	6.57
	Wuhua	2,557	7.05		Guangrao	854	2.35
Guangxi	Shangsi	474	1.31	Shanxi	Hongtong	1,014	2.80
	Zhaoping	530	1.46		Wuxiang	306	0.84
Guizhou	Dafang	1,687	4.65	Shaanxi	Liquan	571	1.57
	Xingren	999	2.75		Yang	530	1.46
Henan	Dancheng	2,375	6.55	Sichuan	Gulin	1,201	3.31
	Qinyang	785	2.16		Wusheng	1,041	2.87
Hubei	Dawu	962	2.65	Yunnan	Lufeng	1,134	3.13
					Luxi	1,762	4.86
Hunan	Chenxi	901	2.48	Chongqing	Fengdu	956	2.64
	Hengshan	444	1.22		Fengjie	1,606	4.43

To help providing a deeper understanding of the survey findings, the study team conducted on-site field studies in eight counties (randomly selected from the 35 counties) in April 2018. The research activities included interviewing directors of education bureaus in each county, on-site visit of schools, interviewing principals and teachers of the visiting schools. Directors in each county were asked about the distribution of schools and teachers within their county, teachers’ wage and benefits in general, teacher recruitment and turnover, teacher rank promotion, etc. 111 principals were asked basic information about the school, the profiles of the teaching staff, the well-being of their staff, and the effect of teachers’ well-being on their work performance and stability. 375 teachers were asked about aspects of their lives and work, such as wage, living conditions, workload, job satisfaction, etc.

Because these interviews were especially meant to get a deeper understanding of the findings of the large-scale teacher questionnaire, insights from these interviews will be brought in in the discussion section below, in which we reflect on the results of the questionnaire survey.

This study was approved by the Academic Review Board of Faculty of Education at the Northeast Normal University, who is in charge of research study ethnics. Before the survey, a research team member gave a standard study introduction to the participants. The introduction included a description of the study and related ethical information, such as confidentiality and the freedom to enrol and quit the study. Due to the fact that this was an online survey, the participants had ample time to consider whether to participate in this survey. The survey did not start unless the participant gave oral consent. The need for consent was waived by the Academic Review Board of Faculty of Education at the Northeast Normal University.

### Measures

The teacher survey questionnaire was divided into two parts, including fixed-response and Likert-style questions. The first part of it comprised questions to obtain information about teachers demographics (e.g., age, gender, marital status), professional information (e.g., education, major, rank, workload), and living conditions (e.g., income, accommodation, insurance coverage, self-perceived social status). The second part of the questionnaire included questions to measure participants' attitudes towards work in terms of job satisfaction, self-perceived social status, turnover intention, working stress, burnout, self-perceived school climate, and teaching efficacy. All Likert-style questions were first translated from existing and validated English questionnaires (specified below) into Chinese by two independent professionals in the field of educational psychology. This questionnaire was then back-translated into English by two professional translators to check for differences between the Chinese version and the original questionnaire. After a careful review, several changes had been made with respect to cultural adaptation and the final version of the questionnaire was provided. After development, the authors together with other researchers executed a pilot study on primary schools teachers in Dongfeng county, Jilin Province and the survey instrument was subsequently modified on the basis of their remarks, prior to use on a large scale. Pre-test participants were excluded from the formal survey.

#### Demographic questions

Teachers reported on their age, gender, family composition (i.e., married, children), working place (county, town, or village schools) as well as hometown location (provincial capital, prefecture-level city, county seat, town seat, or village).

#### Professional questions

Teachers reported on their years of teaching experience, numbers of grades, subjects as well as class hours taught, employment status, initial and final diploma or degrees received, initial and final majors studied, subject(s) taught currently, rank, years spending to the current rank from the previous one, etc..

#### Living condition questions

Teachers reported on their salary, compensation (i.e., health insurance, pension, house allowance), as well as accommodation.

#### Attitudes towards work questions

*Job satisfaction*. We measured teachers' job satisfaction by means of three items. An example of an item is: "I look forward to working in the school every day." Responses were given on a 5-point scale from "Strongly Disagree" (1) to "Strongly Agree" (5). For each participant, four item responses were averaged to create a scale score, ranging from 1 to 5 with higher scores indicating higher degree of job satisfaction. Cronbach's alpha for the scale is .77. Alpha values of .70 or higher are considered to indicate a satisfactory internal consistency [32].

*Self-perceived social status*. We use the MacArthur Scale of Subjective Social Status developed by Goodman et al. [33] to assess teachers’ self-perceived social status. The scale presents the respondent a ladder format with 10 steps. The respondent needs to choose his/her position on the ladder to represent his/her own social status. The highest step represents the highest subjective social status (marked as 10 points) and the lowest step represents the lowest subjective social status (marked as 1 point).

*Turnover intention*. Teachers' turnover intention was measured using two items by Klassen and Chiu [34] which identify thoughts of quitting the teaching occupation. An example of one item is: “I often intend to quit my current teaching profession.” Responses were given on a 5-point scale from "Strongly Disagree" (1) to "Strongly Agree" (5). The scale score was computed as the average item response with a higher score indicating a higher degree of occupational withdrawal intention. Cronbach's alpha was .76 in the present study.

*Job stress*. Teachers’ job stress was assessed using Boyle et al.'s [35] *Teacher Stress Inventory*. Two kinds of job stress were measured: workload stress and students’ behaviour stress. Each type of stress was measured by five items. All items were presented with the question, “As a teacher, how great a source of stress are these factors to you?” and the participants were asked to rate their own level stress on item content such as “responsibility for pupils (e.g., exam success)” (Workload Stress) or “noising pupils” (Students’ Behaviour Stress), with responses ranging from “No stress” (1) to “Extreme stress” (9). Cronbach’s alphas were .75 and .92, respectively, for the two types of stress.

*Burnout*. Burnout was measured using 9-item Bergen Burnout Inventory (BBI-9), which was found to be valid in the education sector [36]. In contrast to the widely used 22-item Maslach Burnout Inventory—Educators Survey (MBI) [37], this scale helped us to end up with a questionnaire of reasonable size. This short Bergen Burnout Inventory has three subscales that are used to evaluate the three domains of burnout: emotional exhaustion (three items), cynicism (three items), and inadequacy at work (three items). Examples of items are: "I often sleep poorly because of the circumstances at work" (emotional exhaustion), "I feel dispirited at work and I think of leaving my job" (cynicism), and "I frequently question the value of my work" (inadequacy at work). A six-point Likert scale was used, ranging from 1 (*to a very low degree*) to 6 (*to a very high degree*). Each subscale score was computed as the average of item responses with higher scores indicating a higher level of burnout. Cronbach's alphas for emotional exhaustion, cynicism, and inadequacy at work were: .75, .84, and .79.

*Self-perceived school climate*. Teachers’ perception of the school climate was assessed using the Revised School Level Environment Questionnaire (SLEQ), which proved to be a reliable and valid tool for studying teachers’ perceptions of school climate [38]. Five dimensions of teachers’ perception of school climate variables were assessed: collaboration (six items), student relations (four items), school resources (four items), decision making (three items), and instructional innovation (three items). Examples of items are: “I have regular opportunities to work with other teachers” (Collaboration), “Students in this school are well behaved” (Student Relations), “Video equipment, tapes, and films are readily available” (School Resources), “Teachers are frequently asked to participate in decisions” (Decision Making), and “New and different ideas are always being tried out” (Instructional Innovation). Responses were given on a 5-point scale from “Strongly Disagree” (1) to “Strongly Agree” (5). The negatively worded items (e.g., “Classroom instruction is rarely coordinated across teachers”) were reversely coded so that a high value indicates the same type of response on every item. For each dimension, the responses were computed as the average of item responses so that high scores indicated strongly feeling of Collaboration, positive Student Relations, high degree of Decision Making, sufficient School Resources, and high degree of Instructional Innovation. Cronbach’s alphas for the five dimensions were .77, .91, .71, .57, and .83, respectively.

### Statistical analysis

Data were analysed to address the three objectives of the study described in the introduction part. To study teachers’ demographic and professional characteristics as well as their living conditions in terms of salary, benefits, and accommodation, we made use of percentiles P10, P25 (first quartile), P50 (the median), P75 (third quartile), and P90. In addition, we examined the variation of these characteristics across different school locales (i.e., county, town, and village) and geographical regions (i.e., eastern, central, and western). Second, teachers’ attitudes towards work were examined by calculating the scale means and standard deviations. Third, we calculated intra-class coefficient (ICC) of these scales to examine the variation of attitudes towards work across schools. All statistical analyses were performed with SAS version 9.3 (SAS Institute Inc., Cary, North Carolina).

## Results

### Demographic characteristics

#### General demographic characteristics

The general demographic characteristics of the study participants are summarized in Table 3. Approximately 67% of the study participants were women. Based on recent national education statistics [16], responding participants were very similar to the entire primary teaching workforce in county areas, which includes 69.2% female teacher. The age of participants ranged from 19 to 68 years and half of the participants were age 38 or younger. Over 88% of responders were married or had previously gone through marriages and 84% had children. Approximately 28% of teachers were working in county schools, 30% in town schools, and 42% in village schools. Over half of the responders’ hometowns were in villages, 23% in town seats, approximately 19% were in county seats, and less than 3% were in cities. This result indicates that almost all county-level primary teachers come from the county areas.

**Table 3:** General demographic characteristics.

	N (%) or Median (P25, P75) N = 36,274
Age	38 (31, 46) years
Gender	
Male	11,945 (32.93)
Female	24,329 (67.07)
Marital Status	
Unmarried	4,218 (11.63)
Married	30,805 (84.92)
Divorced or Widowed	1,251 (3.45)
Number of children	
No	5,750 (15.85)
One	18,921 (52.16)
Two	10,641 (29.34)
More than two	962 (2.65)
Working place	
County	10,302 (28.40)
Township	10,791 (29.75)
Village	15,181 (41.85)
Hometown	
Provincial capital	227 (0.63)
Perfecture-level city	776 (2.14)
County seat	6,754 (18.62)
Town seat	8,336 (22.98)
Village	20,181 (55.63)

#### Age

**Fig 1:**
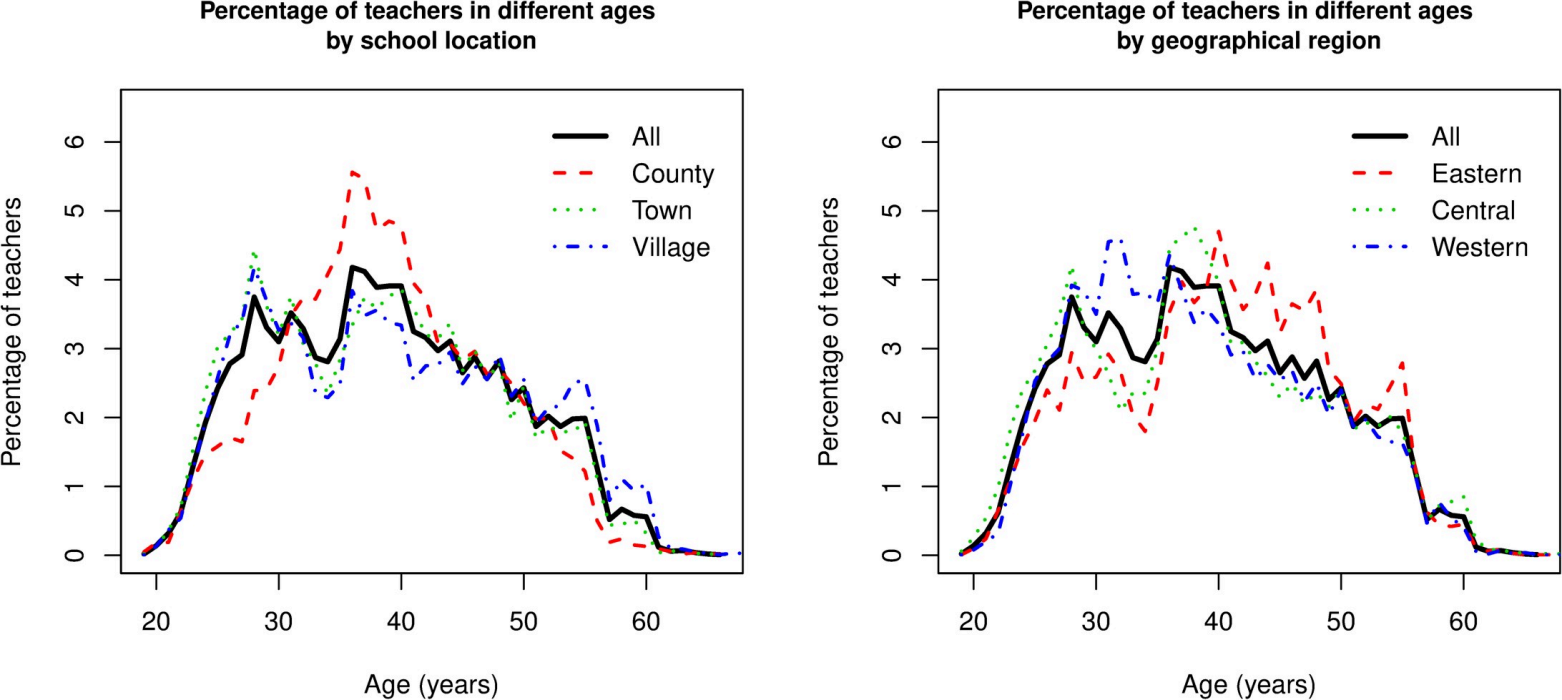
Percentage of teachers in terms of age by school location and geographical region.

With respect to school location, village teachers were older (M = 39.5, SD = 10.15) than those in county schools and in town schools (M = 38.3, SD = 9.44). In addition, as shown in Fig 1, the age distribution among the three school types appeared in different patterns. For instance, town and village schools had higher percentage of teachers under 30 than county schools, while county schools had higher percentage of teachers in the 30-to-40 age range than those in town and village schools. The age distribution also depends on the geographical region (Fig 1). Teachers in the west were the youngest (M = 38.3, SD = 9.20) compared with the counterparts in eastern region (M = 40.3, SD = 9.20) and central region (M = 38.6, SD = 9.77). In eastern provinces, only 23.9% of teachers were in the 25-to-34 age range, compared to 29.1% in central provinces and 36.4% in western provinces. The reverse pattern was found in the 45-to-54 age range, with eastern provinces having the highest proportion, 28.2%. The central and western provinces all had 21.6% of primary teachers in the 45-to-54 range.

#### Gender

**Fig 2:**
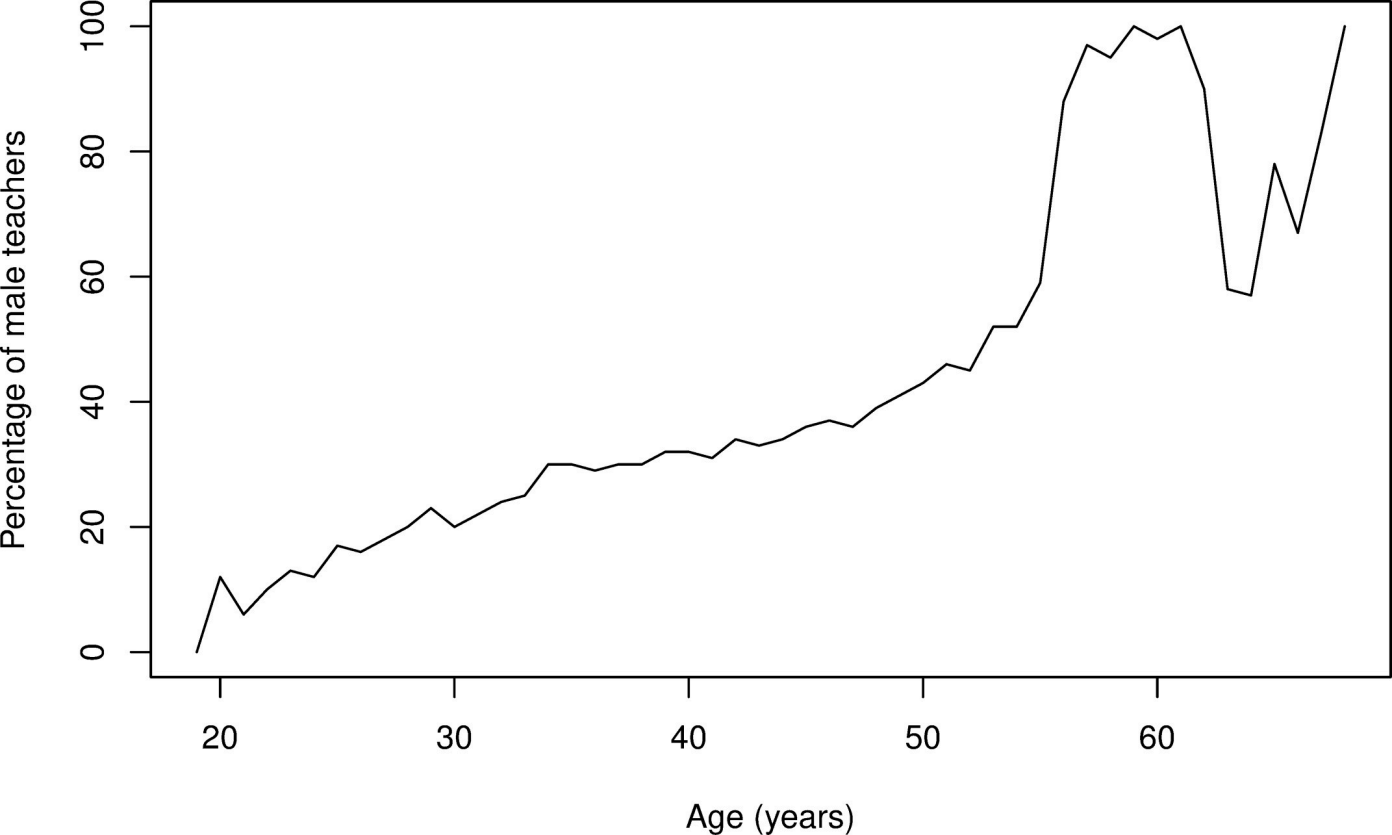
Percentage of male teachers by age.

Fig 2 suggests that the proportion of male teachers is gradually changing: the younger the age, the lower the proportion of male teachers. The two variables, age and proportion of male teachers, were strongly correlated, r(48) = 0.90, p < .0001. The mean age of male and female participants was significantly different, t(36,272) = 58.79, p < .0001; on average, female participants were younger (M = 37, SD = 8.65) than their male counterparts (M = 43, SD = 9.69). Regarding the whole male teacher population, the proportion of teachers at age 30 or younger accounted for only 10%, reflecting the lack of attractiveness of teaching occupation in county areas to young males.

**Fig 3:**
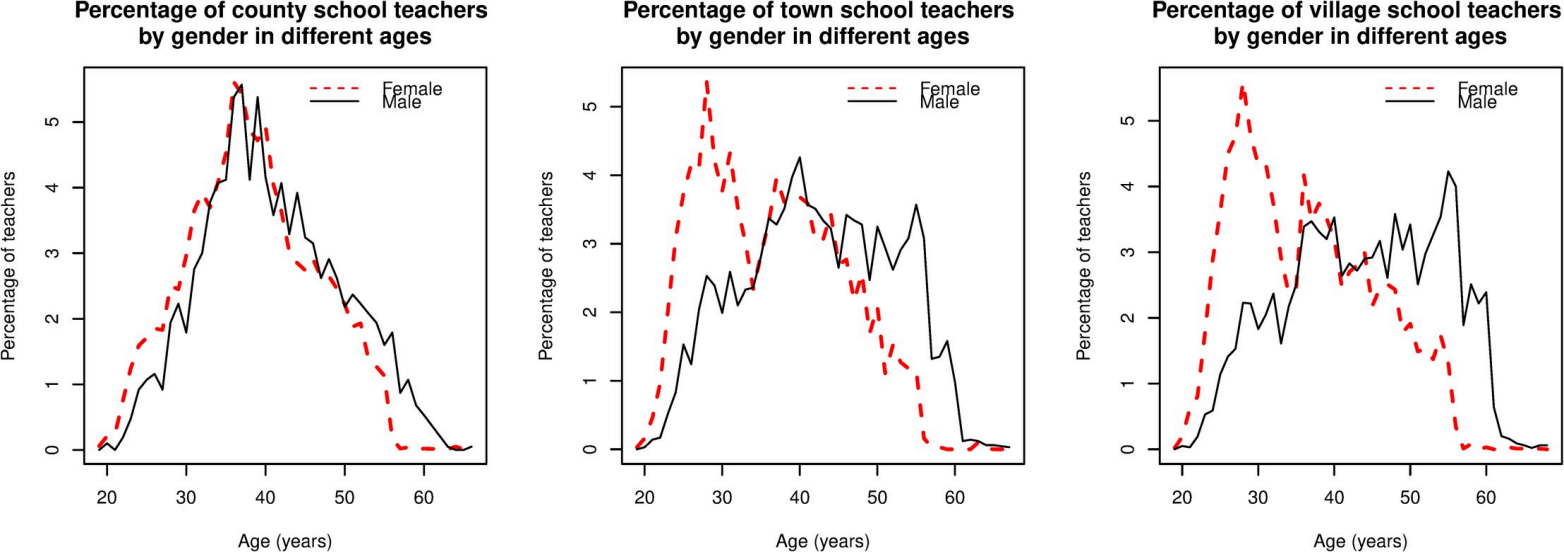
Percentage of male and female teachers in terms of age in county, town, and village schools.

There was considerable variation in the proportions of male and female teachers across different school locations and geographic regions. Female teachers account for 80%, 68%, and 57% of the number of teachers in county, town, and village schools. This seems to indicate that the ratio of male and female teachers is more balanced in village schools. However, after taking age into account, we found a more nuanced story. As Fig 3 shows, the age distributions of male and female teachers in county schools were approximately bell-shaped. The modal ages for male and female teachers were 37 and 36. As to town and village teachers, as Fig 3 also shows, the modal ages of female teachers were both 28, while the modal age of male teachers was 39 and 56. This result indicates that, on one hand, male teachers in town and village schools are considerably older than female teachers, and older than male teachers in county schools; on the other hand, most of young female teachers were teaching at town and village schools. It can be concluded that in village schools (and to a smaller extent in town schools), the teaching workforce is mainly composed of older male teachers and young female teachers.

Table 4 illustrates the proportion of male and female teachers across the three geographical regions. The percentage of female teachers in primary schools ranged from 62.7% in Western China, over 68.5% in Central China, to 72.2% in Eastern China. This may reflect that the position of primary school teacher is considered less attractive for men in the eastern region than for men in the western and central regions.

**Table 4:** Distribution of the teachers by geographical region and gender.

	Gender		
	Male *n* (%)	Female *n* (%)	Total *n* (%)
Eastern	2,691 (27.83)	6,980 (72.17)	9,671 (26.66)
Central	3,585 (31.48)	7,802 (68.52)	10,791 (31.39)
Western	5,669 (37.26)	9,547 (62.74)	15,261 (41.95)
Total	11,945 (32.93)	24,329 (67.07)	36,274 (100)

### Professional characteristics

#### General professional characteristics

Table 5 presents professional characteristics of the total sample. Respondents reported a median of 19 years of experience as a primary school teacher. About 28% of participants reported that they taught two or more grades simultaneously. A large proportion of teachers (73%) in the sample taught multiple subjects. A majority of teachers (87%) reported that they were in a permanent employment position. Teachers possessing a senior rank title accounted for only 3.5%. In addition, the median number of years before achieving a senior rank title was 10 years, which was much longer compared with the promotion time for lower rank titles.

**Table 5:** Professional characteristics of the total sample.

	N (%) or Median (P25, P75)
Years of teaching	19 (7, 27)
Number of grades taught	
One grade	22,497 (62.0)
Two grades	8,464 (23.3)
Three grades or more	5,313 (14.7)
Number of subjects taught	
One subject	9,785 (27.0)
Two subjects	9,075 (25.0)
Three subjects	7,031 (19.4)
Four subjects or more	10,383 (28.6)
Class hours per week	16 (13, 20)
Employment status	
Permanent	31,550 (87.0)
Temporary	2,133 (5.9)
Free pre-service students	444 (1.2)
Special teaching post plan students	1,721 (4.7)
Others	426 (1.2)
Initial diploma when starting teaching career	
Bachelor degree or higher	5,686 (15.7)
Associate degree	10,218 (28.2)
Normal school diploma	16,208 (44.7)
High school diploma	3,517 (9.7)
Junior middle school diploma or lower	645 (1.8)
Highest diploma earned	
Bachelor degree or higher	19,577 (54.0)
Associate degree	14,718 (40.6)
High school/vocational school/normal school diploma or lower	1,979 (5.5)
Rank of teachers	
Senior teacher	1,256 (3.5)
Class 1 teacher	11,533 (31.8)
Class 2 teacher	11,702 (32.3)
Class 3 teacher	7,518 (20.7)
Not yet ranked (due to being newly appointed)	4,276 (11.8)
Years spending to the current rank from previous one	
Senior teacher	10 (8, 14)
Class 1 teacher	8 (5, 12)
Class 2 teacher	5 (3, 9)
Class 3 teacher	3 (1, 3)

#### Teaching experience

**Fig 4:**
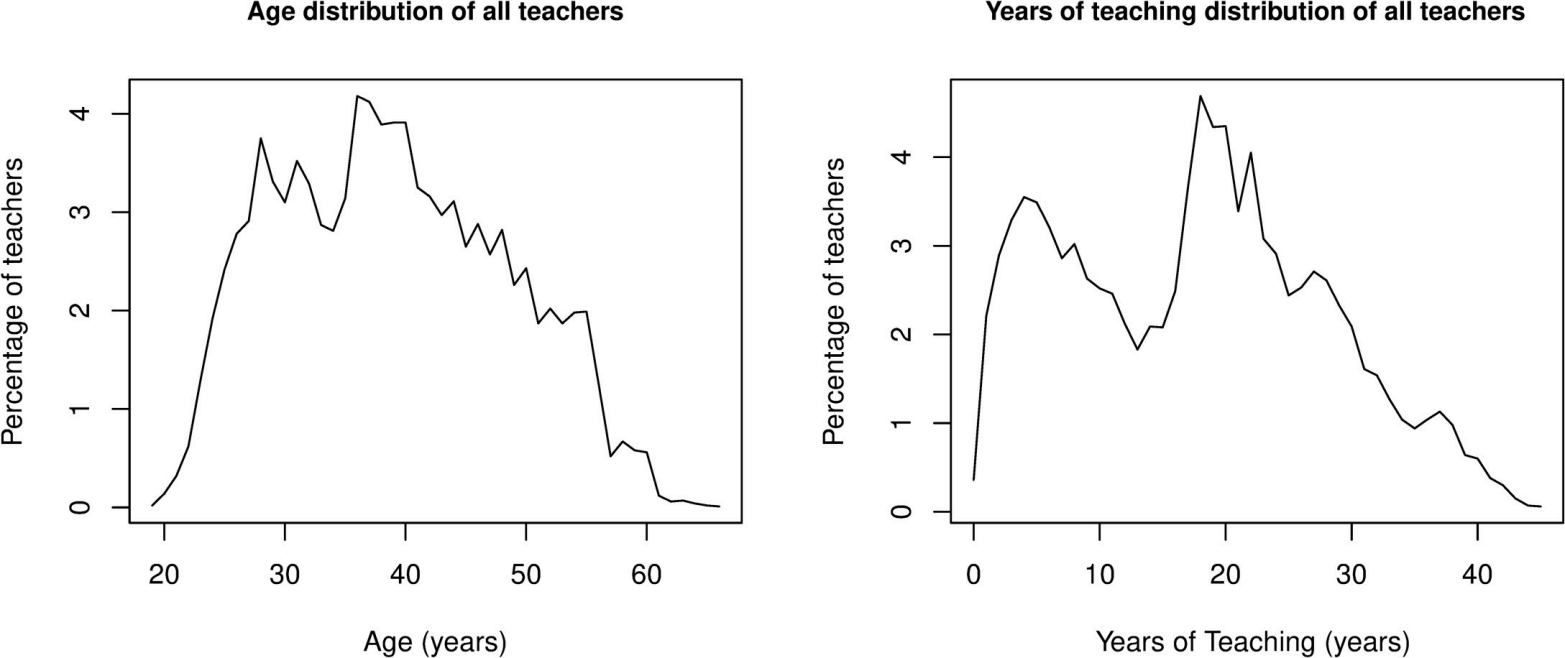
Percentage of teachers in terms of age and years of teaching.

As shown in Fig 4, teachers’ age and teaching experience have a similar distribution pattern, although the distribution of teaching experience is somewhat more bimodal (with peaks around 5 and 20 years of experience). In fact, there was a strongly positive correlation between the two variables, r(36,272) = 0.92, p < .0001. As a result, the distribution patterns of years of teaching and age across school location and geographical region were very similar as well. For instance, in village schools, almost 41% of male teachers had taught for more than 25 years, while 43% of female teachers had less than 10 years of teaching experience. This result reaffirmed the unbalanced age and gender composition among the teaching workforce in village schools.

#### Educational background

Educational background can serve as an indicator of the quality of teaching workforce [13]. As seen in Table 5, approximately half of participants held a normal school diploma when starting their teaching career and only less than 16% with a bachelor or higher degree. It is noted that normal schools were at upper-secondary education level, preparing teachers for primary schools and kindergarten and were the lowest level institutions of Chinese pre-service teacher education system before 1999 [39]. When asked about their highest diploma earned, approximately 95% of the respondents reported holding an associate degree or higher. A majority of county-level teachers obtained higher degrees during their teaching career through a variety of in-service college or university programs such as radio and TV lectures and correspondence courses (we refer to Buley-Meissner [40], and Zhou and Reed [27] for the history, forms, and contents of these programs).

An examination of initial education background of teachers by age range (see Table 6) revealed that approximately 90% respondents between 25 and 34 held either an associate (48.8%) or bachelor degree (41%) as their initial degree, which was far greater than those of old age range. For instance, only 12% of respondents aged between 45 and 54 had an initial associate or bachelor degree. This result showed the quality improvement of the teaching workforce.

**Table 6:** Initial diploma by age, school location, and geographic regions.

	Initial diploma				
	Junior secondary school	Senior secondary school	Normal school	Associate degree	Bachelor degree or above
Age					
< 25	0.3	0.6	4.2	62.0	32.8
25–34	0.2	1.1	8.8	48.8	41.0
35–44	0.9	4.3	70.5	20.3	4.1
45–54	4.4	22.3	60.6	12.2	0.6
>55	6.1	43.6	42.8	6.9	0.7
School location					
County	0.5	4.2	53.9	27.5	14.0
Town	2.0	7.9	44.2	28.3	17.6
Village	2.5	14.7	38.7	28.6	15.5
Geographical regions					
Eastern	3.2	10.0	47.8	26.2	13.0
Central	0.9	10.3	45.1	27.5	16.2
Western	1.6	9.1	42.4	29.9	17.0

#### Major studied

The participants were also asked to report their initial major when starting their teaching career and the final major (See Table 7). As to the initial major studied, the top five majors with the highest proportions were general teacher education program in normal schools, other majors that were not relevant to the main subjects taught in the primary schools, Chinese, English, and mathematics. It is noted that the proportions of participants, whose majors were music, art, physical education, and informatics, only accounted for 13.2% in total. However, the proportions of participants who taught art, music, physical education (PE), and informatics lessons were 20.3%, 21.8%, 22.3%, and 9.5%, which indicated big shortage of professionally trained music, art, PE, and informatics teachers. As to the final major studied, the top five majors with the highest proportions were Chinese, other majors, English, pedagogy, and mathematics. By comparison with the first major, we found that most of the teachers chose Chinese, pedagogy, and ideological and political education as a major to pursue their highest diploma.

**Table 7:** Distribution of first and final major of the participants.

First major	N (%)	Final major	N (%)
Chinese	5,142 (14.2)	Chinese	15,606 (43.0)
Mathematics	2,603 (7.2)	Mathematics	2,697 (7.4)
English	2,985 (8.2)	English	3,001 (8.3)
History	143 (0.4)	History	317 (0.9)
Geography	74 (0.2)	Geography	124 (0.3)
Physics	173 (0.5)	Physics	189 (0.5)
Chemistry	316 (0.9)	Chemistry	302 (0.8)
Politics	261 (0.7)	Politics	581 (1.6)
Biology	199 (0.6)	Biology	166 (0.5)
Music	1,137 (3.1)	Music	829 (2.3)
Art	1,274 (3.5)	Art	1,001(2.8)
Physical Education	1,423 (3.9)	Physical Education	1,070 (3.0)
Pedagogy	948 (2.6)	Pedagogy	2,708 (7.5)
Psychology	94 (0.3)	Psychology	89 (0.3)
Informatics	995 (2.7)	Informatics	742 (2.1)
General teacher education	12,922 (35.6)	General teacher education	2,540 (7.0)
Others	5,585 (15.4)	Others	4,312 (11.9)

#### Workload

Respondents taught a median of 16 class hours (one class hour equal 40 to 45 minutes depending on different regions) during a regular teaching week. In terms of average class hours taught by school location, village teachers taught the most (M = 19, SD = 5.9), followed by teachers in town schools (M = 15.2, SD = 4.5). Respondents in county schools taught the fewest number of hours (M = 14, SD = 3.7). An examination of average class hours taught per week by geographical regions revealed that respondents from the central region (M = 16.8, SD = 5.6) and the west tended to teach the most (M = 16.8, SD = 5.5). Respondents who worked in the east tended to teach relatively less (M = 15.4, SD = 5.1).

#### Rank and promotion period

Teachers were also asked to report their current ranks. Table 7 showed that 32.3% of the respondents had rank Level 2. This was followed by 31.8% of respondents who were Level 1 teachers. Only 3.5% of respondents were ranked as Senior and 20.7% of respondents reported themselves as Level 3 teachers. There were 11.8% of respondents still in probationary status. An examination of ranks by school location revealed that of respondents who worked in county and town schools, 4.3% and 4.2% were senior teachers, while only 2.4% of respondents in village schools were senior teachers. In terms of geographic regions, 5.4% of respondents from the east reported themselves senior titled teachers, followed by 3.0% in the west. Respondents who worked in the central region had the lowest proportion of senior teachers, 2.5%. In addition, teachers from the west had the largest proportion of Level 3 and not yet ranked teachers accounting for 37.6%, while the proportion of the same group was approximately 29% in the eastern and central regions. This result is due to the high proportion of young teachers in the west.

Results showed the median time prior to promoting to each of the four ranks were: three years (Level 3), five years (Level 2), eight years (Level 1), and ten years (Senior), indicating more time is spent on obtaining higher rank. In terms of school location, the median time spent for a senior rank was 10 years for county teachers and 11 years for both township and village teachers. Teachers from three school locations had the same medians of promotion time: eight years, five years, and three years. In terms of geographic regions, teachers from the west spent more time for promotion to higher ranks. Specifically, half of western teachers needed to spend 12 years, 10 years, and 6 years to reach the Senior, Level 1, and Level 2 title, while the time spent for eastern teachers were 10 years, 7 years, and 5 years. The results indicated rank promotion for western teachers takes longer compared with the eastern and central regions.

### Living state characteristics

#### General living state characteristics

On average primary teachers in all county-level schools earned approximately 3,800 yuan per month. 92.4% of teachers reported that they enjoy benefits such as pension, unemployment insurance, medical insurance, and housing fund. 68.8% of respondents had their own housings, 14.3% lived in schools’ apartments or dorms, while the rest 16.9% rent an apartment or lived with others. Of respondents who indicated they lived in schools, only 18% of those were satisfied or very satisfied with schools’ accommodation.

#### Salary and salary satisfaction

Table 8 shows several key statistics on the income of the total sample, by school locations, by geographic regions, and by intervals of years in teaching career.

**Table 8:** Teachers’ income by total sample, school location, geographic region, and intervals of years in teaching career.

				Percentile				
	*N*	Mean	SD	10	25	Median	75	90
All	34,783	3,773.4	1124.7	2,500.0	3,000.0	3,680.0	4,500.0	5,300.0
County	9,999	3,618.1	1027.0	2,478.0	2,990.0	3,500.0	4,200.0	5,000.0
Township	10,411	3,799.3	1114.8	2,510.0	3,000.0	3,680.0	4,562.0	5,300.0
Village	15,181	3,862.6	1184.1	2,500.0	3,000.0	3,800.0	4,700.0	5,500.0
Eastern	9,419	4,257.2	1172.7	2,750.0	3,400.0	4,275.0	5,000.0	5,800.0
Central	11,036	3,208.4	932.8	2,160.0	2,609.5	3,084.0	3,743.0	4,500.0
Western	14,328	3,890.5	1040.7	2,708.0	3,100.0	3,800.0	4,508.0	5,300.0
<1–5	6,955	2,948.7	795.0	2,000.0	2,471.1	2,900.0	3,450.0	4,000.0
6–10	4,488	3,413.0	873.2	2,402.0	2,800.0	3,400.0	4,000.0	4,600.0
11–15	2,792	3,570.0	897.0	2,600.0	2,980.0	3,500.0	4,104.5	4,859.0
16–20	5,837	3,605.7	918.8	2,610.0	3,000.0	3,450.0	4,199.0	5,000.0
21–25	4,734	3,991.3	969.4	3,000.0	3,300.0	4,000.0	4,600.0	5,329.0
>25	9,977	4,561.9	1121.1	3,100.0	3,886.0	4,600.0	5,300.0	6,000.0

Incomes are expressed in Chinese *yuan* (1 US dollar equals to approximately 7 Chinese yuan). 1491 missing values on income were excluded.

Depicted in Table 8, respondents working in rural schools reported the highest mean and median incomes, followed by in township school. Teachers working in county schools had the lowest mean and median income.

From the perspective of regional difference, there is a large variation of income among teachers from different geographical regions. The median of teachers’ income in the east was the highest at 4,275 yuan per month, the median of income was 3,800 yuan per month in the west, and teachers in the central region had the lowest median of income at 3,084 yuan per month. Using percentiles for further analysis, we found that the 10^th^ percentiles of income, 2,750 yuan and 2,708 yuan in the eastern and western regions were even higher than the first quartile of income in the central region, 2,609 yuan. The proportion of teachers with income above 4,000 yuan per month is 60% in the east, 40% in the west, and only 20% in the central region.

### Characteristics of attitudes towards work

#### General characteristics of attitudes towards work

Table 9 shows the mean and standard deviation of the scores of all teachers for each scale or subscale of working characteristics. At the same time, we reported the intra-group correlation coefficient (ICC) of scores on the scales or subscales, calculated using multilevel linear regression models, which can be interpreted as the percentage of variance in scores among teachers that can be attributed to differences between schools.

**Table 9:** Scores on scale variables of attitudes towards work and variance explained at school level.

	Mean	Standard deviation	ICC
Job satisfaction	3.51	0.90	17.9%
Perceived social status	3.80	2.07	15.6%
Turnover intention	2.09	0.92	10.1%
Burnout			
Emotional exhaustion	4.12	1.23	12.5%
Cynicism	2.79	1.37	12.5%
Inadequacy	3.25	1.42	11.5%
Stress			
Workload	7.46	1.57	8.1%
Student Management	5.74	2.05	10.3%
School climate			
Teacher cooperation	3.73	0.67	11.1%
Student relations	3.85	0.78	13.1%
School resources	3.17	0.79	28.4%
Decision making	3.10	0.56	8.3%
Instructional innovation	3.81	0.74	10.2%

#### Score variation among schools

In the last column of Table 9, the percentage of explained variance at school level (ICC) in the unconditional model is presented for each scale or subscale. The values of ICCs ranged from 4.7% (classroom management) to 28.4% (school resources), indicating the proportion of the variance for each scale variable explained by differences among schools. It is striking that the variance in scores for the scales “job satisfaction”, “self-perceived social status”, “turnover intention”, stress for “student management”, and some subscales of “school climate” and “burnout” can be attributed for more than 10% to differences between schools.

Table 10 shows the summary statistics for these variables, splitting up according to the school location. Compared with teachers working in town and village schools, on one hand, teachers from county schools were less satisfied with their job, felt lower social status, had higher turnover intention, were identified higher level of emotional exhaustion, cynicism, and inadequacy, and felt higher pressure on student management. For example, approximately 26% of teachers in county schools fell below the overall 20^th^ percentile in the job satisfaction score, compared to 17% in village schools. On the other hand, teachers working in county schools perceived better atmosphere of teacher cooperation and more sufficient school resources than teachers in town and village schools. Specifically, approximately 33% of teachers in county schools scored above the overall 80^th^ percentile in the school resources score, compared with approximately 25% and 18% in town and village schools.

**Table 10:** Scores on selected scale variables of attitudes towards work by school location.

	N	Overall mean (SD)	% below the overall 20th percentile	% above the overall 80th percentile
Job satisfaction, *F*(2,36271) = 396.45, *p*<.0001				
County	10,302	3.31 (0.92)	25.8	18.3
Township	10,791	3.52 (0.89)	20.4	24.7
Village	15,181	3.63 (0.88)	17.1	29.4
Perceived social status, *F*(2,36271) = 44.27, *p*<.0001				
County	10,302	3.64 (1.96)	0.20	0.16
Township	10,791	3.84 (2.09)	0.19	0.19
Village	15,181	3.89 (2.12)	0.19	0.21
Turnover, *F*(2,36271) = 77.28, *p*<.0001				
County	10,302	2.17 (0.92)	22.9	30.8
Township	10,791	2.09 (0.92)	26.8	27.3
Village	15,181	2.03 (0.92)	29.9	25.1
Exhaustion, *F*(2,36271) = 60.41, *p*<.0001				
County	10,302	4.23 (1.23)	13.5	25.8
Township	10,791	4.12 (1.23)	15.8	22.5
Village	15,181	4.06 (1.24)	17.1	21.2
Cynicism, *F*(2,36271) = 143.54, *p*<.0001				
County	10,302	2.96 (1.40)	15.0	20.0
Township	10,791	2.81 (1.36)	17.3	16.8
Village	15,181	2.67 (1.34)	20.1	14.3
Inadequacy, *F*(2,36271) = 83.98, *p*<.0001				
County	10,302	3.39 (1.43)	17.3	17.3
Township	11,791	3.26 (1.43)	19.8	15.7
Village	15,181	3.15 (1.40)	21.4	12.9
Student management, *F*(2,36271) = 214.22, *p*<.0001				
County	10,302	6.01 (2.04)	14.9	21.8
Township	11,791	5.83 (2.01)	16.7	18.4
Village	15,181	5.49 (2.05)	21.3	14.5
Teacher cooperation, *F*(2,36271) = 81.69, *p*<.0001				
County	10,302	3.8 (0.68)	21.0	28.1
Township	11,791	3.71 (0.66)	23.2	23.2
Village	15,181	3.70 (0.66)	22.8	22.3
Student relations, *F*(2,36271) = 11.70, *p*<.0001				
County	10,302	3.85 (0.78)	23.4	20.9
Township	11,791	3.83 (0.77)	23.9	19.1
Village	15,181	3.88 (0.78)	22.4	21.8
School resources, *F*(2,36271) = 741.51, *p*<.0001				
County	10,302	3.37 (0.75)	18.6	32.6
Township	11,791	3.22 (0.75)	25.0	25.2
Village	15,181	3.00 (0.82)	35.5	18.4

All categories of teachers seemed on average to feel high workload, were not satisfied with decision making at schools, and have high confidence in their capabilities on students’ engagement, class management, and instructional strategies, regardless of school locations.

## Discussion

We report here a comprehensive, national study of the demographic and professional characteristics, living conditions, and attitudes towards work of Chinese primary teachers in county areas. The responding sample of nearly 37,000 teachers represents the largest study of Chinese primary teaching workforce ever reported. Strengths of the survey are its high response rate, its representative nature and its very large sample size, making that this study not only gives a detailed insight in the teachers’ workforce, but also can serve in the future as a baseline measurement to evaluate progress.

The results show that the primary teaching workforce is becoming dominantly female. Of concern when looking across the age range of respondents, is the marked reduction in male primary teachers that enter the teaching workforce in the last 15 years. The current age distribution of male and female teachers provided important evidence of future gender imbalances in the teaching workforce. For instance, women made up 88% of teacher under the age of 25, while they accounted for 58% of those in the 45 to 54 age group. Through the oral interviews, we learned that a significant reason for men’s reluctance to become primary school teachers was due to the low salary. Young males often preferred other higher-paid occupations. To become a primary school teacher is more attractive to women. Female teachers interviewed indicated that the stability of the teaching profession and adequate holidays are the main attractions for them to become teachers. The data also shows that a higher proportion of male teachers in the western region than that in the eastern region. With limited opportunities in the private sector, a primary school teacher position, which implies the prospect of a pension, comprehensive medical insurance and security of tenure, is still appealing to the males in the western region (as also remarked by Smith [41]). Further strategies for attracting males into the primary teaching workforce need to be taken into account.

The age distribution of teachers in county schools followed a more symmetric unimodal distribution, but the age distribution in village schools was similar to a “dumbbell” age structure. The large increase in young teachers in village schools can be attributed to a series of important policies introduced by the Chinese government to improve rural education in recent years. For example, *the Special Post Teacher Plan (SPTP)* policy was launched in 2006 to recruit university graduates to work for three years in rural schools in China's Central and Western provinces at the cost of central government finance, with an emphasis on remote minority regions and educationally disadvantaged counties. In addition to these national policies, local county governments have also taken measures to balance the distribution of teachers at all levels of schools. For example, many of the counties researched require newly hired teachers to teach in town or village schools for the first three years of their employment, which, we believe, is the key reason for the large number of young female teachers in village schools. The large number of older male teachers, though with many years of teaching, does not mean they are experienced teachers. Some of them only had a lower secondary school diploma when starting their teaching career. Many principals during the survey confirmed that some older male teachers had insufficient subject matter knowledge and adopted outdated teaching methods to teach.

The “dumbbell” age structure implies the extreme lack of key and experienced teachers and the incompleteness of the teaching workforce, which may lead to severe negative consequences to teachers, students, and schools. Firstly, many young teachers in village schools may be lack of teaching experience and expertise and they need expert teachers to help them develop instructional skills. However, with very few veteran teachers, novice village school teachers seldom have good mentors to learn from, which is detrimental to their professional growth. Secondly, we found that, although allocated to teach in villages, a significant number of young teachers transferred to other schools in or close to county seats upon completion of the first three-year teaching. As a result, students in village schools are more likely to encounter a string of underprepared or inexperienced teachers, thus experiencing a cumulative effect that is much more damaging to their learning than one year of poor teaching would create [42]. Thirdly, the shortage of expert teachers may jeopardize the “collective knowledge” of a school, which leads to inadequacy in supporting sound educational decision or collegial learning [42]. A rational system of teacher rotation is needed to safeguard the improvement of the academic performance of village students, the professional development of novice teachers, and the adequacy of “collective knowledge” in village schools.

The proportion of teachers with an initial associate or bachelor degree in the younger age cohort was much higher than those in old age groups, which may serve as an indicator of teacher quality improvement in recent years. For instance, the share of teachers with at least an associate degree was 94% under the age of 25, while the proportion was only 25% in the age group between 35 and 44. In terms of final education degree, approximately 95% of respondents had an associate or bachelor degree. For those with an initial diploma from normal schools or high schools, they generally obtained higher degrees via correspondence study, self-study examination, and TV universities [27]. Although witnessing a large improvement of teachers’ education attainment, we still need to maintain a clear mind. The data showed that a majority of teachers chose Chinese, pedagogy, or political sciences as their majors for advanced training and studies since these disciplines were easy to follow, while very few pursued a degree on high-demand subjects such as mathematics and English. This may lead to a hidden shortage of qualified teachers in these subjects.

Although educational degree is an indicator necessary to be considered for teaching qualification, teachers who teach content areas must also have appropriate content knowledge and expertise. However, we found a widespread practice of out-of-field teaching in county-level primary schools. Out-of-field teaching refers to the fact that teachers are assigned to teach subjects for which they have no formal trainings and qualifications [43]. For instance, among the teachers who taught mathematics, the proportion of teachers with an initial major in mathematics only made up 14%, teachers with normal schools background accounted for 29%, and the rest 57% of teachers had the expertise in Chinese, English, art, music, etc. The out-of-field teaching problem was even worse in the subjects such as art, music, and physical exercises. Teachers majoring in art only made up 3.1% of the total sample. Among the art teachers, only 11% had art expertise and 27% with normal schools background. We observed 12.3%, 7.6%, 5.8% of arts teachers having initial major in Chinese, mathematics, and English, respectively. The out-of-field teaching may lead to very bad effects such as damaging the quality of teaching, disrupting the school schedule, and increasing burden on existing teachers [44]. Some provinces began to take some actions against this kind of hidden teacher shortage. For instance, in Jiangxi province, senior college students majoring in music, art, and physical education were encouraged to take their internship in township and village schools to guarantee the in-field teaching of these subjects.

On average, teachers in village schools can earn 250 yuan more than those in county schools. This may be attributed to the national policies on promoting rural teacher development in recent years. For instance, in order to improve the compensation of village school teachers, *The Rural Teachers Support Plan (2015–2020)* was promulgated in 2015 by the State Council aiming at attracting new teachers and retaining current teaching in town and village schools through a list of measures, such as raising salaries and subsidizing new graduates. Our results showed that less than 10% of teachers were satisfied or very satisfied with their salary. In order to have an accurate assessment of teachers’ salary level, we need to compare teacher salaries with salaries of other occupations in the county-level labour market. Unfortunately, there are no good comparative data available for national samples. We will make a crude comparison through the content of teacher interviews. Most oral interviewed teachers, especially teachers in township and village schools, recognized that increase in wages in recent years and gave high appraisal for local governments’ effort. However, compared with other occupations (in particular civil servants, another large workforce financed by county government), teachers salary was relative low. Some teachers mentioned that there was no big difference in nominal income between teachers and civil servants. But civil servants could enjoy extra subsidies for transportation and telecommunication plus a merit-based bonus by the end of the year, which may reach up to multiple times the teachers’ monthly salary. How teachers’ actual salaries are aligned with the civil servants workforce will be an important issue for the local government.

There was considerable regional variation in teachers’ salary. This result is consistent with Wang and Lei’s [45] findings concerning regional imbalance of teachers’ salary. On average, teachers in Eastern China earned 1,000 yuan more than those working in Central China. Since 2001, China has begun to establish a “county-centered” system to implement the compulsory education. Under this system, the county-level people’s governments are the major administrator and provider of compulsory education and the salaries of teacher will be paid directly by county-level financial department [46]. Since most east coastal areas are richer than the central and western areas, it is obvious that salary in the east is higher than the other two areas. Besides, as the poorest areas in China, western provinces have been implemented preferential policies (e.g., extra financial subsidies) by the central government, which greatly relieved the fiscal pressure on education investment for the county government. Under this background, western counties have the abilities to invest more money on the teachers’ salary since some education investment such as textbook fees and school construction can be subsidized by the central or provincial government. For the central area, almost all the education investments depend on the county fiscal level, which leads to the inadequacy of investment on teachers’ salary. Further policies and measures needs to be promulgated to alleviate this regional imbalance on teachers’ salary.

Teachers in county-level schools perceived a low social status in general, with an average score of 3.8 in a 10-point scale. This may be attributed to low salary, difficult career development, and deceasing prestige. Teachers’ perceived social status varies between different school locations. Teachers working in county schools perceived the lowest social status, while village school teachers had the highest average social status score. When asked why they felt a low social status, teachers from county schools mentioned that besides low salary, they enjoyed lower prestige compared with counterparts from other occupations, such as civil servants. As to teachers from township or village schools, due to the implementation of policies such as rural teacher subsidies and rural teacher support programs in recent years, their salary had a big increase. In addition, teachers traditionally had higher prestige and influence in villages. All these made teachers feel higher social status.

The data also provided some encouraging findings on teachers’ working characteristics. Firstly, the majority of teachers had very low turnover intention, which can serve as an indicator of the stability of teaching workforce; secondly, the average score of cynicism was low indicating that teachers still had much enthusiasm towards the teaching career and their students regardless of heavy workload and low pay; thirdly, teachers had strong beliefs in their ability on student engagement, class management, and instructional strategies, which may lead to high teaching effectiveness, abundant instructional practices, and the improvement of students’ academic achievement [47].

Several areas of working characteristics need improvement. In general, teachers had a moderate level of job satisfaction, felt heavy pressure from workload, which accompanied with high degree of emotional exhaustion. Besides, they experienced senses of insufficient school resources and low participation in decision making. In terms of school location, teachers in counties schools had the lowest job satisfaction, highest degree of emotional exhaustion, and highest level of stress from workload. While village teachers felt more satisfied with their job and had lower level of emotional exhaustion and stress from workload. However, they experienced a sense of lack of school resources the most.

### Study limitations

Although this study presents a broad view of primary teaching workforce in county areas, a major limitation is its cross-sectional nature. The teaching workforce is constantly changing and needs continuous evaluation. Yet, this study can serve as the baseline data for a longitudinal teaching workforce study that gathers data about county-level primary teachers periodically allowing for an investigation of trends over time. A second limitation of this study is that we had to make a selection in the variables to be measured, and certain relevant variables (e.g., on teachers’ numeracy and literacy abilities) were not included. Future research should consider investigating the difference between urban and county-level teachers with respect to numeracy and literacy abilities and the trends over time so that an evaluation on the effectiveness of policies such as *The Rural Teachers Support Plan (2015–2020)* can be achieved. Another area for research is investigating the association between demographic, professional, living variables and constructs regarding working characteristics. In order to achieve this goal, further multivariate analyses such as SEM need to be conducted so that an overall picture of the relationship among these variables and constructs can be depicted. Finally, research should also consider the impact of teachers’ characteristics on students’ academic achievement, which can provide further implications for future policy making.
